# Metastatic Parotid Myoepithelial Carcinoma in a 7-Year-Old Boy

**DOI:** 10.1155/2012/212746

**Published:** 2012-09-13

**Authors:** Issam Saliba, Nazir El Khatib, Antoine Nehme, Selim Nasser, Nabil Moukarzel

**Affiliations:** ^1^Sainte-Justine University Hospital Center (CHU Sainte-Justine) and Department of Pediatric Otolaryngology Head & Neck Surgery, Montreal University, Montreal, QC, Canada H3T 1C5; ^2^Department of Otolaryngology Head & Neck Surgery, Sacré-Coeur Hospital, Lebanese University, Beirut, Lebanon; ^3^Department of Pathology, Sacré-Coeur Hospital, Beirut, Lebanon

## Abstract

Myoepithelial carcinoma is a rare malignancy of the parotid gland that is usually seen in adults. We report the first case in children of myoepithelial carcinoma of the parotid gland with massive invasion of the facial nerve and metastasis to cervical lymph nodes. Due to its rarity, the treatment and the clinical course of this tumor are not well defined yet. We performed a total parotidectomy, a modified neck dissection, and a postoperative radiotherapy in 7-year-old boy. Sparing of the facial nerve was impossible; it was sacrificed and grafted with a sural nerve. Histopathology confirmed the diagnosis of a parotid gland carcinoma and immunohistochemical markers showed that the tumor cells express cytokeratin, epithelial membrane antigen, cytokeratin 7, smooth muscle actin, P63, CEA, and S100. This pattern of immunostaining is consistent with the diagnosis of myoepithelial carcinoma. On the postoperative tenth month he presented with a pulmonary and lumbar vertebra metastasis.

## 1. Introduction

Sheldon was the first to identify myoepithelial salivary gland tumor as a distinct neoplastic entity in 1943 [1] and it was first described by Stromeyer et al. in 1975 [2]. Coupled with the rarity of this lesion, the diagnosis is further complicated by the considerable variability in morphologic features and clinical prognosis. Myoepithelial carcinoma has been included in the World Health Organization classification of salivary gland tumors since 1991 [3]. Myoepithelial carcinoma of the parotid gland represents less than 5% of all salivary gland tumors. Due to its rarity, the treatment and clinical course of this tumor are not well defined yet. We report the first case of myoepithelial carcinoma of the parotid gland in children with massive invasion of the facial nerve and metastasis to cervical lymph nodes.

## 2. Case Presentation

A 7-year-old boy was referred to our department for the evaluation of a mass in the left upper cervical region. The child described a painless tumor that had been progressively increased in size for the last six months without any associated symptoms. The child was known to be healthy, vaccinated without any specific medical problems. Physical examination showed a soft, deep, and immobile, noninflammatory, 3.0 × 2.5 × 2.0 cm left parotid mass associated with left facial paresis grade III (House-Brackmann classification). Multiple left upper cervical enlarged lymph nodes were noticed. The largest one reaching 2.0 × 2.0 × 2.0 cm was hard and mobile. 

**Figure 1:**
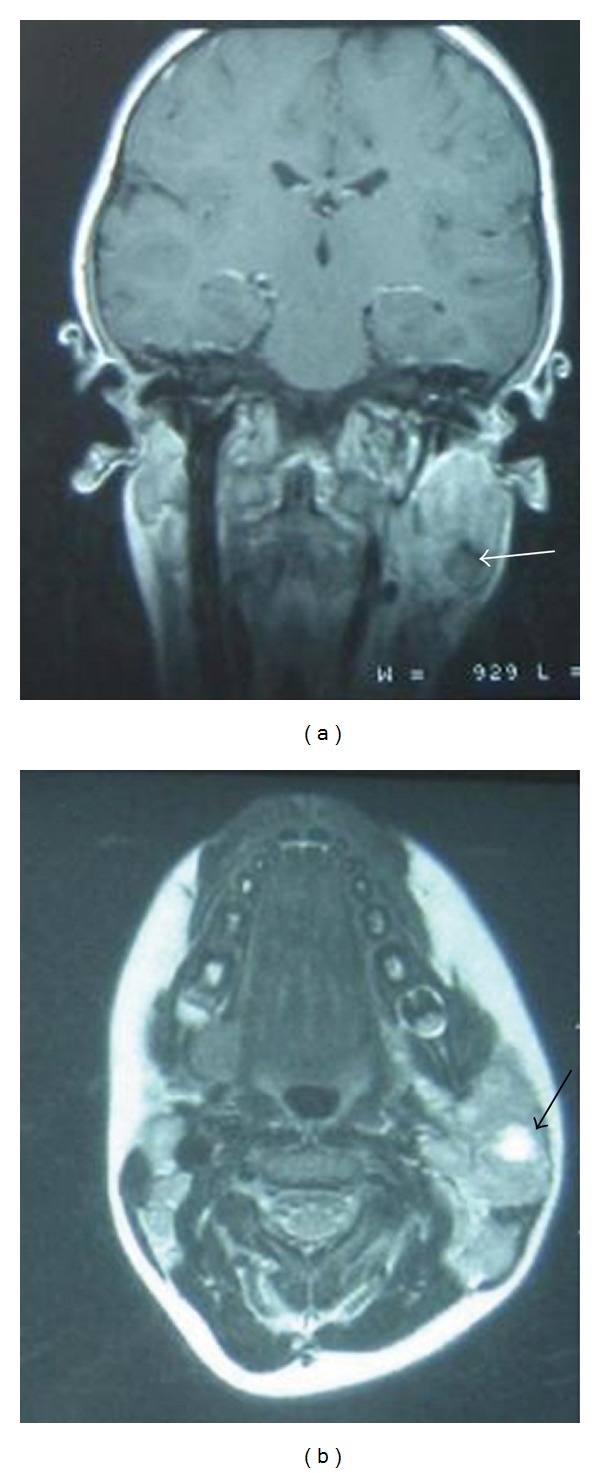
Coronal T1-weighted (a) and axial T2-weighted (b) cervical magnetic resonance imaging showing a parotid gland tumor with a heterogeneous signals with a central area of necrosis (arrow).

The remaining otolaryngology exam was within normal limits. Serologies for Epstein-Barr virus, cytomegalovirus, *Bartonella henselae* as well as tuberculin PPD test (purified protein derivative) were all negative. Cervical magnetic resonance imaging (MRI) (Figure 1) showed a large tumor with heterogeneous signals of left parotid gland with multiple necrotic areas, associated with multiple jugulodigastric and retrocervical lymph nodes. A fine-needle aspiration (FNA) revealed atypical cells without a definite diagnosis; open biopsy of the cervical lymph nodes showed a metastatic carcinoma consistent with myoepithelial carcinoma of salivary gland origin. Additional workup including chest X-ray, brain MRI, and a computerized tomography (CT) scan of the abdomen were within normal limits. Subsequently the patient was planned for surgery. 

Left total parotidectomy with modified neck dissection of the cervical areas I, II, III, and IV was performed. The tumor was of 3.0 × 2.5 × 2.0 cm lobulated, dark red color invading the superficial and deep lobes of the parotid gland. The main trunk of the facial nerve and its subdivisions were totally invaded by the tumor. Thus sacrificing the facial nerve was necessary for a one-block excision of the tumor. A left sural nervous graft of 8 cm, with two branches (Y shape), was grafted between the main trunk proximally and the mandibular as well as the zygomatic branch distally. The accessory nerve, internal jugular vein, and the sternocleidomastoid muscle were preserved. A frozen section of the parapharyngeal fat was negative for malignancy.

The patient had an uneventful postoperative course and was discharged home on the fourth postoperative day with a total left facial nerve paralysis.

**Figure 2:**
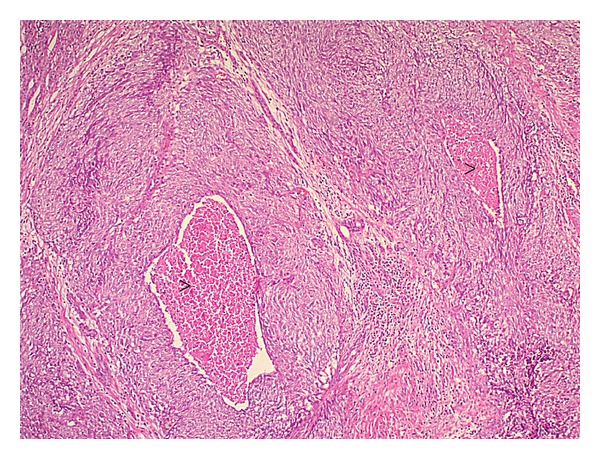
Low-magnification view of representative histopathology field of the lesion. Tumor shows epithelioid and spindled cells with central zone of necrosis (Arrow heads). (H&E 10x).

Histopathology showed a 3.0 × 2.5 × 3.0 cm high-grade myoepithelial carcinoma (Figure 2) replacing most of the gland, extending to the extraglandular tissues, invading the facial nerve with lymphatic vascular invasion. The margins were free of tumor. Metastatic carcinoma was present in 7 of 14 periglandular nodes and in 6 of 47 left cervical nodes. 

The tumor was composed of nests and sheets of spindled and epithelioid cells with areas of necrosis. Immunostains showed that the tumor cells express cytokeratin, EMA, cytokeratin 7, smooth muscle actin, P63, CEA, and S100 and are negative for desmin, LCA, CD34, and CD20. This pattern of immunostaining is consistent with the diagnosis of myoepithelial carcinoma.

Because of the aggressiveness of this tumor, the patient underwent postoperative radiotherapy, despite the lack of clear guidelines in the literature. MRI and whole body positron emission tomography (PET) scan performed six months later were normal. Unfortunately, on the postoperative tenth month, the patient presented a pulmonary and lumbar vertebra metastasis.

## 3. Discussion 

Myoepithelial carcinomas are usually known as a soft tissue tumor all over the body. Salivary glands myoepithelial carcinomas occur in adults at a mean age of 55 years [4]. To our best knowledge, only one case of myoepithelial carcinoma of the parotid gland has been reported in children [5]. However, we report the first case of parotid myoepithelial carcinoma which is associated to a massive invasion of the facial nerve, to a cervical lymph nodes metastasis and complicated by a pulmonary and lumbar vertebra metastasis in a 7 year-old boy.

Pediatric salivary gland neoplasms are malignant in 33% of cases compared to 20% of adult salivary gland tumors [6]. Female-to-male ratio is 2 : 1. In contrast to adults, the larger the gland is, the higher the likelihood of malignancy in children is. Therefore, 85% of salivary gland malignancies found in children originate in the parotid gland [7]. Thus a child who presents with a firm parotid mass should be suspected of harboring a malignancy. 

Malignant salivary gland neoplasm's usually present as a painless swelling but they are more frequently symptomatic than benign lesions: facial nerve paresis (10 to 15%), pain (10 to 29%), and fixation of the mass to the underlying structures are the most presented symptoms. They usually indicate local or regional tumor extension [8]. 

MRI is well known to be the preferred imaging study to evaluate salivary gland masses. It shows the margins of the tumor more sharply than does the CT scan. MRI is especially helpful in case of facial nerve infiltration where it gives an abnormal enhancement of the invaded segment and an increase in nerve diameter [9].

PET scan is more sensitive (86%) but less specific (75%) than either CT or MRI (57% sensitivity and 92% specificity) in detecting micrometastasis and in differentiating tumor from postirradiation changes [10–13]. In addition, PET scanning still lacks resolution to define margins of involvement so it is of little use in assessing tumor extension. 

Fine-Needle Aspiration (FNA) is a minimally invasive procedure. Reports of FNA in children are encouraging, citing minimal discomfort and no need for general anesthesia [14]. It may allow rapid diagnostic and obviate the need for open biopsy. In cases of parotid mass where FNA is inconclusive, the minimal procedure for diagnosis and treatment of a solitary parotid mass should be a superficial parotidectomy with facial nerve sparing. 

The histological spectrum of salivary gland neoplasms in the pediatric age group is similar to that of the adult population. However, the incidence of the different types is not the same. Myoepithelial carcinoma constitutes less than 2% of salivary gland carcinoma. Distinction between malignant and benign myoepitheliomas may sometimes be difficult. The relative lack of cytological atypia distinguishes these tumors from myoepithelial carcinomas [4]. Originally classified as mixed tumors, the majority of myoepithelial carcinomas develop in a pleomorphic adenoma [7]; in these cases they are mainly low-grade malignancies [15]. When they appear in isolated form or de novo, as in our case, the carcinoma is often high grade [16]. Immunohistochemical studies of myoepithelial carcinomas show that these tumors usually express epithelial markers (cytokeratin and epithelial membrane antigen) and to varying extent markers of smooth muscle differentiation such as calponin (75%) and smooth muscle actin (50%). Other markers are expressed in varying degrees: S-100 protein (100%), Vimentin (100%), and gliofibrillary protein acid (31%). The most sensitive myogenic marker in a series of 29 myoepithelial carcinoma of soft tissues was calponin (positive in 100% of the cases), but this antibody has little specificity, as it is also expressed in other tumors showing smooth muscle or myofibroblastic differentiation [4]. Seethala et al. found the recently developed antibody P63 to be the best myoepithelial marker [17]. 

Owing to its rarity, there are yet no clear guidelines for the management of myoepithelial carcinoma. For localized salivary gland tumors, wide surgical excision is the mainstay of therapy, and adjuvant radiation therapy with or without cervical lymph node dissection is frequently preformed [4, 18]. The use of radiation therapy in combination with surgery has improved the locoregional control and survival rate for patients with major salivary glands carcinoma [19, 20]. The issue of postoperative radiation therapy in the pediatric population is controversial, in light of the inherent risk to develop a second malignancy. Complications related to radiation therapy are not trivial, with one study reporting a 60% rate of sequelae such as dental caries, prolonged trismus, facial deformity, and osteoradionecrosis. Postoperative radiation therapy is suggested for high-grade malignancy, microscopic residual tumor, perineural invasion, soft-tissue extension, or positive lymph nodes in multiple levels and after salvage surgery. Few clinical reports are found in the literature with respect to adjuvant chemotherapy for malignant salivary neoplasms and especially myoepithelial carcinoma [4]. 

Regarding facial nerve involvement, there was only mild weakness in our case despite the massive tumor invasion similar to the other reported case [5]. This might be due to the persistence of an intact intratumoral nerve fibers. Because high-grade malignancies are extremely uncommon in children, preservation of the facial nerve should be the rule unless it is invaded by the tumor. However there is yet no consensus regarding sparing or not of the facial nerve in case of myoepithelial carcinoma [7, 21, 22]. Parents should always be counseled regarding the risk of facial nerve injury and the need to sacrifice the nerve if the intraoperative findings suggest tumor invasion or the preoperative biopsy confirms a high-grade malignancy. Perineural spread may occur in an axial and a circumferential pattern along the involved nerves; retrograde tumor spreading allows the tumor to reach the temporal bone and a skull base invasion. 

The prognosis of malignant salivary neoplasm in the pediatric population depends on the tumor type and grade. In the most common salivary gland malignancy (Mucoepidermoid Carcinoma), relapse rates for high-grade tumors are 30% to 50% after a parotidectomy and enucleation, respectively [7]. According to Terhaard et al., facial nerve paralysis secondary to salivary neoplasms is associated with high incidence of regional and distant metastases [23]. Factors suggesting a poor prognosis are facial palsy, pain, rapid tumor growth, and the presence of ipsilateral lymph node enlargement in the cervical region. However, an ordinary-appearing clinical presentation could be falsely reassuring. 

Overall, myoepithelial carcinomas in children seem to have a somewhat more aggressive clinical course than those in adults. Aggressive treatment as well as close and prolonged clinical and radiological followup are recommended regarding the aggressiveness of the tumor and its unknown behavior. Careful case assessment must include an attention to clinical presentation, intraoperative findings, and histopathologic features to ensure that the correct diagnosis is established.
